# Synthesis and Characterization of a Heteroleptic Ru(II) Complex of Phenanthroline Containing Oligo-Anthracenyl Carboxylic Acid Moieties

**DOI:** 10.3390/ijms11093158

**Published:** 2010-09-08

**Authors:** Adewale O. Adeloye, Peter A. Ajibade

**Affiliations:** Department of Chemistry, Faculty of Science and Agriculture, University of Fort Hare, Private Bag X1314, Alice 5700, South Africa; E-Mail: aadeloye@ufh.ac.za

**Keywords:** polypyridyl ligands, ruthenium complex, oligoathracene, palladium, conjugation, spectroscopy, electrochemistry

## Abstract

In an effort to develop new ruthenium(II) complexes, this work describes the design, synthesis and characterization of a ruthenium(II) functionalized phenanthroline complex with extended π-conjugation. The ligand were L_1_ (4,7-bis(2,3-dimethylacrylic acid)-1,10-phenanthroline), synthesized by a direct aromatic substitution reaction, and L_2_ (4,7-bis(trianthracenyl-2,3-dimethylacrylic acid)-1,10-phenanthroline), which was synthesized by the dehalogenation of halogenated aromatic compounds using a zero-valent palladium cross-catalyzed reaction in the absence of magnesium-diene complexes and/or cyclooctadienyl nickel (0) catalysts to generate a new carbon-carbon bond (C-C bond) polymerized hydrocarbon units. The ruthenium complex [RuL_1_L_2_(NCS)_2_] showed improved photophysical properties (red-shifted metal-to-ligand charge-transfer transition absorptions and enhanced molar extinction coefficients), luminescence and interesting electrochemical properties. Cyclic and square wave voltammetry revealed five major redox processes. The number of electron(s) transferred by the ruthenium complex was determined by chronocoulometry in each case. The results show that processes **I**, **II** and **III** are multi-electron transfer reactions while processes **IV** and **V** involved one-electron transfer reaction. The photophysical property of the complex makes it a promising candidate in the design of chemosensors and photosensitizers, while its redox-active nature makes the complex a potential mediator of electron transfer in photochemical processes.

## 1. Introduction

Ruthenium(II) polypyridyl complexes have attracted attention in recent years due to their well-defined spectroscopic, photophysical, photochemical and electrochemical properties [1,2]. These properties are of particular use in the construction of supramolecular systems [3] and in the development of photochemically driven molecular devices [4]. Ruthenium(II) polypyridyl complexes have also received attention as functional models for water-oxidation catalysis in photo-system and photochemical cleavage of water [5,6]. Turning the optical properties of transition metal complexes by ligand tailoring is a fascinating research field which has generated highly coloured pigments, very efficient triplet energy and electron transfer reactions, long-lived excited states, charge separated species, and singlet oxygen producers [1]. Along these lines, polyaromatic modified bipyridines and phenanthrolines have extensively been studied and interesting features connected to lifetime enhancement from the excited state manifold has been reported [7–9]. Ru(bpy)_3_^2+^ has certainly been one of the molecules most extensively studied and widely used in research laboratories during the last 10 years due to its chemical stability, redox properties, excited state reactivity, luminescence emission, and excited state lifetime [10]. In particular, the Ru(II) polypyridine complexes have played and are still playing a key role in the development of photochemistry, photophysics, photocatalysis, electrochemistry, photoelectrochemistry, chemi- and electrochemi-luminescence, and electron and energy transfer [4,11,12]. Improvement of the photophysical properties of polypyridyl-based Ru(II) complexes is currently the object of intensive studies [13–17]. It was shown [18] that one of the best way to enhance both the absorption coefficient and red-shift of the metal-to-ligand charge transfer (MLCT) band in a ruthenium-based photosensitizer was to extend the π-conjugation length of the colorant’s ancillary [19] or anchoring [20] ligands.

Existing design strategies for the preparation of these advanced multicomponent molecules follow two principal methods. One approach exploits the use of metallosynthons bearing reactive functions such as triflates, halides and carbonyls as the intermediates for producing the final species with the expected features. In another approach, the ligand is first constructed and subsequently coordinated to the appropriate metal. In both cases, the quest for new and alternative approaches for easy building and organizing various photoactive partners around photoactive metals is one of the main aims of this field [21]. Some authors reported the synthesis of heteroleptic ruthenium complexes by extending the conjugation length of the ancillary ligand [22,23]. Such heteroleptic ruthenium complexes have a strong MLCT band and dye solar cells devices based on them display very good photovoltaic performance. In spite of this, the main drawback of these sensitizers is the lack of absorption in the red region of the visible spectrum and also relatively low molar extinction coefficient [24]. Many researchers have tried to overcome these shortcomings without significant success [25–28]. The molecular engineering of ruthenium complexes for TiO_2_-based solar cells presents a challenging task as several stringent requirements have to be fulfilled by the sensitizer and these are very difficult to be met simultaneously, including absorption of all the visible light and function as an efficient charge transfer sensitizer. The aim of the present study is to synthesize a heteroleptic ruthenium(II) complex that can be used in energy and electron transfer processes, dendrimers for light harvesting and lightpowered molecular machines.

## 2. Results and Discussion

### 2.1. Syntheses

The strategy developed in this contribution was prompted by the fact that we were able to find a satisfactory solvent system combination to overcome the poor solubility property of 9,10-dibromoanthracene in common organic solvents, and a subsequent coupling reaction with 4,7-dibromo-1,10-phenanthroline. The traditional method of producing polymerized hydrocarbon by dehalogenation of halogenated hydrocarbon using biscyclooctadienyl nickel (0) or magnesium-diene complexes as catalyst and the Sonogashira couplings of aryl bromide under palladium-catalyzed cross reaction are well documented [29–32].

**Scheme 1:**
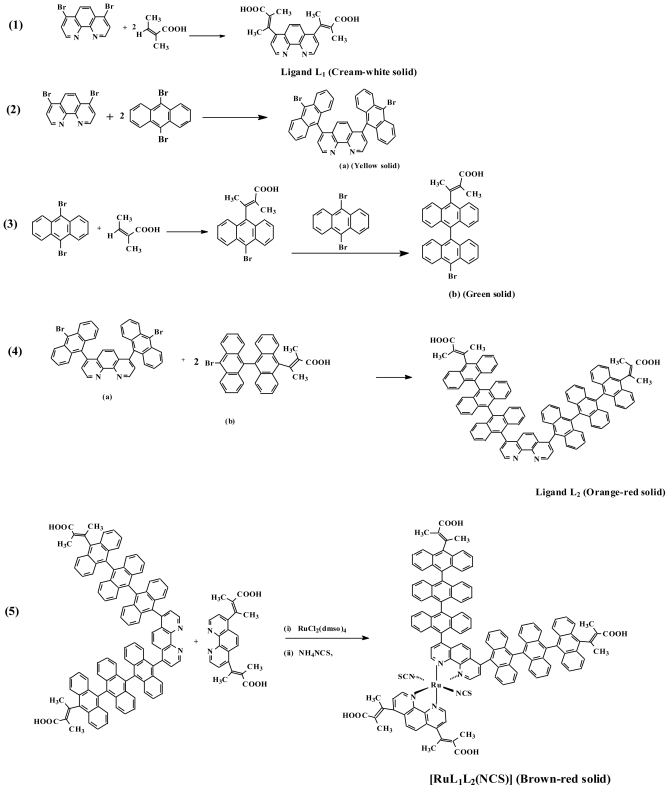
Synthesis of functionalized oligoanthracenyl and alkenyl phenanthroline ligands L_1_ and L_2_ and their Ru(II) complex [Ru-L_1_L_2_(NCS)_2_]. {Reaction conditions: (1) MeOH, Et_3_N, Pd/C, 24 h; (2–4) DCM/Benzene, or EtOH, Et_3_N, Pd/C, 8 h; (5) DMF, 12 h, NaOH/HNO_3_, Diethyl ether}.

Scheme 1 shows the synthetic route for the formation of the ligands 4,7-bis(2,3-dimethylacrylic acid)-1,10-phenanthroline (L_1_) and (4,7-bis(trianthracenyl-2,3-dimethylacrylic acid)-1,10- phenanthroline (L_2_) as well as the ruthenium complex [Ru(*cis*-dithiocyanato-4,7-bis(2,3- dimethylacrylic acid)-1,10-phenanthroline)-4,7-bis(trianthracenyl-2,3-dimethylacrylic acid)-1,10- phenanthroline)]. The ligand L_1_ was synthesized in a one-pot synthetic reaction procedure (Equation 1), while L_2_ was synthesized in three steps (Equations 2–4). A stoichiometric calculation, reaction in 50% dichloromethane-benzene solvent mixture and the use of higher temperature for the synthesis of L_2_ limits the formation of mixture of compounds observed when milder experimental conditions and inappropriate solvents are used. For example, poor product yield and mixtures were obtained when the reactions were carried out in ethanol and palladium(II) acetate. Thin layer chromatography was used during and after reaction to ascertain the formation and purity of the products [33]. The ruthenium complex was synthesized in a one pot synthesis starting from dichlorotetrakis-(dimethyl sulphoxide) ruthenium(II) in DMF following standard literature procedures (Equation 5) [34–36].

### 2.2. Infrared Spectra

**Figure 1:**
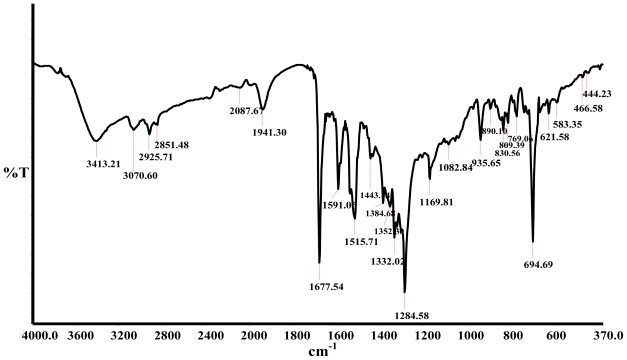
Infra-red spectra of the RuL_1_L_2_(NCS)_2_ complex in KBr pellet.

The infrared spectra of the ligands and the complex were compared and assigned on careful comparison. The bands in the region 3600–2900 cm^−1^ due to the O-H and C-H vibrations in the spectra of the ligands undergo only slight changes, which are ascribed to interaction between the free -COOH groups on the complex and the possibility of intra molecular hydrogen bonding between the free -COOH groups. Figure 1 shows the Fourier Transform Infrared (FT-IR) spectrum of the complex [RuL_1_L_2_(NCS)_2_] in KBr pellet. The complex shows a broad absorption frequency at 2087 cm^−1^ for stretch vibrational modes due to the N-coordinated ν(CN). This band is very close in intensity compared to the band at 809 cm^−1^, due to ν(CS). The bands at 1677 cm^−1^ and 1284 cm^−1^ were assigned to the ν(C=O) and ν(C-O) stretching of carboxylic acid groups, respectively. The three bands at 1591, 1515 and 1443 cm^−1^ are due to ring stretching modes of the ligands. The band at 1384 cm^−1^ was assigned to the carboxylate symmetric ν(−COO^−^) of the carboxylic acid group. A comparison of the infrared spectra of L_2_ and 9,10-dibromoanthracene showed that a strong vibrational band in the former was conspicuously absent in the latter, confirming the loss of C-Br bond and the formation of C-C bond linkages of the polyanthracenyl group. Furthermore, the C-C bond linkage between anthracene and phenanthroline was affirmed by the absorption frequency at 778 cm^−1^. Peaks in the region 770 and 730 cm^−1^ demonstrate the existence of four adjacent hydrogen atoms common to L_2_ and complex [RuL_1_L_2_(NCS)_2_]. All vibrational peaks in the region are found relatively weak and broad in the complex, which may be ascribed to the loss of crystallinity and the broad distribution of the anthracene chain length [37]. The weak absorption frequencies at 466 and 444 cm^−1^, respectively, show the coordination of nitrogen atoms of the ancillary ligands to ruthenium central metal atom [38].

### 2.3. NMR

The ^1^H NMR spectra of ligands L_1_, L_2_ and [RuL_1_L_2_(NCS)_2_] complex are consistent with the structures shown in Scheme 1. Due to the presence of two different anchoring ligand substitutions, such that all the protons are electronically found in different environments, gave complications in the proton NMR spectra of the [RuL_1_L_2_(NCS)_2_] complex. ^1^H NMR spectra of L_1_ showed four doublets and a singlet peak in the aromatic region (δ 9.09–7.72 ppm); these were unambiguously assigned to H-2, H-3, H-8, H-9 and H-5, H-6 respectively. The methyl protons were found as singlet-triplet peaks in the aliphatic region. When L_1_ were compared to L_2_ proton NMR spectra, two additional doublet peaks at δ 7.99 and 7.45 ppm were found, these were assigned to the anthracene protons. All the peaks were shifted downfield in the complex between δ 8.30 and 7.78 ppm. The deshielding pattern is ascribed to the lone pair-lone pair electron donation of the nitrogen atoms to the *d*-orbital of the ruthenium metal. The chemical shifts of the ancillary phenanthroline protons were observed as doublets at reduced intensity [39,40]. In the ^13^C NMR spectra, the carbonyl chemical shifts at δ 169.67, 183.1 and 183.2 ppm were assigned respectively to L_1_, L_2_ and [RuL_1_L_2_(NCS)_2_] complex carboxylic acid function. It is interesting to note that the progressive increase toward the downfield shifts may also be partly due to extension of π-conjugation length in the molecules. The NCS peaks were found at δ 134.9 and 133.6 ppm due to *trans* orientation to other ligands. Carbon-13 NMR spectra of complexes containing NCS ligands are useful in the identification of mode of coordination. N-coordinated thiocyanate carbon resonance peak has been reported in the number of complexes at δ 130–135 ppm [41,42]. Thus, the presence of peaks at 130–135 ppm region in complex [Ru(II)L_1_L_2_(NCS)_2_] indicates that the NCS ligands were coordinated through the nitrogen end [43,44]. The high intensity peaks at δ 134.1 and 127.2 ppm and 128.1 and 125.3 ppm, common to both L_2_ and the [RuL_1_L_2_(NCS)_2_] complex, were assigned to the triply-linked anthracene carbon molecules.

### 2.4. Electronic and Emission Spectra

**Figure 2:**
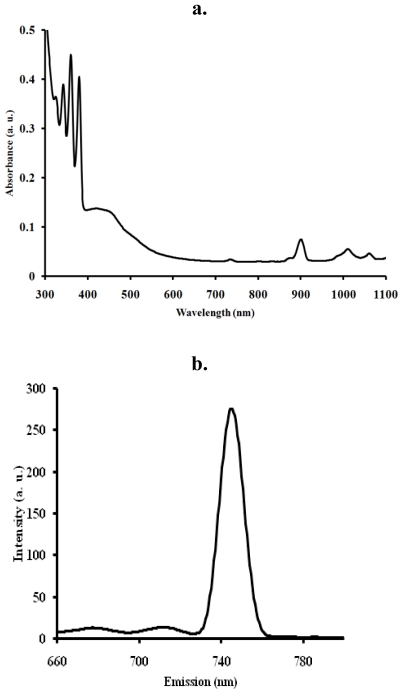

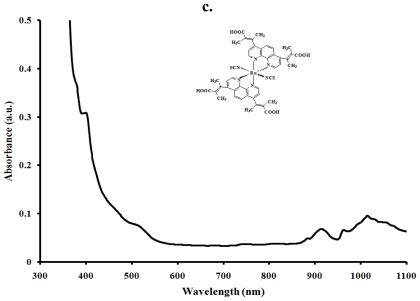
UV-Vis absorption (**a**) and emission (**b**) spectra of the RuL_1_L_2_(NCS)_2_ complex at a concentration of 0.001 g/dm^3^ in chloroform-methanol (1:1). (**c**). UV-Vis absorption spectrum of [Ru(L_1_)_2_(NCS)_2_], showing the effect of conjugation at the visible region of the metal-to-ligand charge transfer transition.

The UV-Vis absorbance and emission spectra of the [RuL_1_L_2_(NCS)_2_] complex in chloroformmethanol mixture (1:1, v/v) are presented in Figure 2a. In the UV- region, the [RuL_1_L_2_(NCS)_2_] complex displays four distinct vibronic peaks for the intra ligand (π→π*) charge transfer transitions characteristics of anthracene derivatives at 318, 324, 360 and 380 nm [45]. The [RuL_1_L_2_(NCS)_2_] complex shows broad and intense absorption band between 400 and 503 nm, due to metal-to-ligand charge transfer transitions (MLCT) [45]. The effect of extending the π-conjugation length through anthracene were observed when [RuL_1_L_2_(NCS)_2_] complex spectrum was compared to a similar [Ru(L_1_)_2_(NCS)_2_] complex with no anthracene substitution (Figure 2b). The molar extinction coefficient of the lowest energy MLCT band in the [Ru(L_1_)_2_(NCS)_2_] complex was ɛ = 7620 M^−1^ dm^−3^, which is ≈45% lower than the [RuL_1_L_2_(NCS)_2_] complex at (λ_max_ = 503 nm, ɛ = 16800 M^−1^dm^−3^). Wu, Shi-Jhang and co-workers have reported preparation of several heteroleptic ruthenium complexes by extending the conjugation length of the ancillary ligands [45–51]. Such heteroleptic ruthenium complexes have a strong MLCT band and DSC devices based on them display very good photovoltaic performances. In the long wavelength tail of the absorption spectrum, small but significantly distinct shoulders at 900 nm (ɛ = 1514), 1007 nm (ɛ = 1082) and 1054 nm (ɛ = 900) were present. These absorption features correspond to the lowest ^3^MLCT excited states [1,52]. Groups which extend the delocalization of the π systems of polycyclic arenes cause further bathochromic shifts, but the extent of these shifts vary with the positions of substitution [53]. Upon excitation into the ^1^LC and ^1^MLCT bands, (λ_exc_ = 470 nm), the RuL_1_L_2_(NCS)_2_ complex displays appreciable luminescence at room temperature (λ_em_ = 745 nm), (Figure 2a). The luminescent properties of a complex as well as its ability to play the role of excited state reactant or product are related to the energy ordering of its low energy excited states and, particularly, to the orbital nature of its lowest excited state. With the choice of ligands, it is well thought that the energy positions of the MC, MLCT, and LC excited states of [RuL_1_L_2_(NCS)_2_] complexes depend on the ligand field strength [1]. The B3LYP/6-31G theoretical calculations showed that the electronic structures of anthracene derivatives are perturbed by the side substitutes on the anthracene block, and the slight variation of the electronic structures results in the enhanced electron accepting ability and the decrease of the HOMO-LUMO energy gap, which is the origin of the shifting of emission wavelength to blue-green region [55].

### 2.5. Electrochemistry

**Figure 3:**
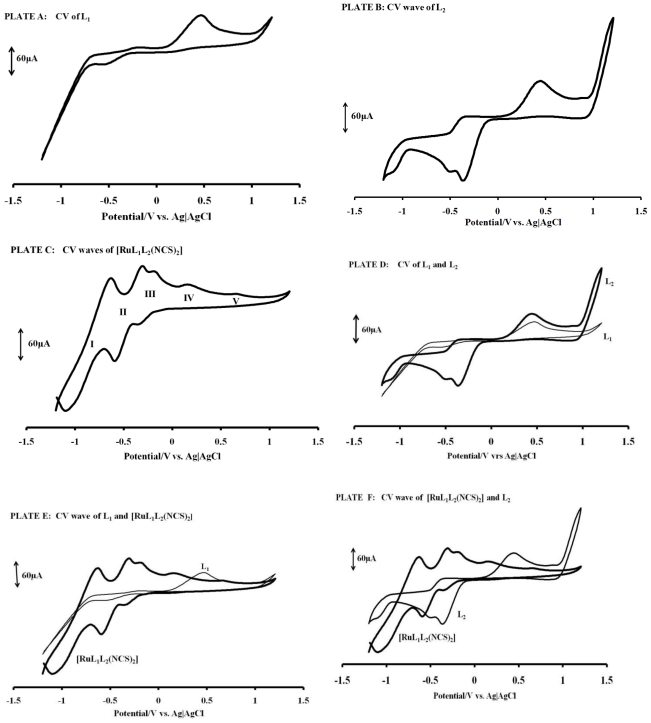
Cyclic voltammetry profiles of 1 × 10^−3^ M of complex L_1_, L_2_ and [RuL_1_L_2_(NCS)_2_] in freshly distilled DMF containing 0.1 M TBABF_4_ supporting electrolyte. Step potential: 5 mV, amplitude: 50 mV *vs*. Ag|AgCl, frequency: 10 Hz. Scan rate: 100 m Vs^−1^ *vs*. Ag|AgCl.

**Figure 4:**
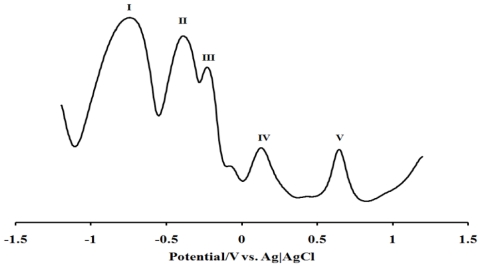
Square wave voltammetry profiles of 1 × 10^−3^ M of complex [RuL_1_L_2_(NCS)_2_] in freshly distilled DMF containing 0.1 M TBABF_4_ supporting electrolyte. Step potential: 5 mV, amplitude: 50 mV *vs*. Ag|AgCl, frequency: 10 Hz. Scan rate: 100 m Vs^−1^ *vs*. Ag|AgCl.

The cyclic voltammograms of L_1_, L_2_ and [RuL_1_L_2_(NCS)_2_] in DMF, containing tetrabutylammonium tetrafluoroborate as supporting electrolyte, are shown in Figure 3 (Plates A–F), with their electrochemical data in Table 1. Figure 4 is the square wave voltammogram of the complex in DMF, containing the same electrolyte. Irreversible oxidation peaks were found at +0.48 and +0.45 V respectively, for L_1_ and L_2_. Other redox waves were observed at E_½_ = −0.41 V (for L_1_) and E_1/2_ = −0.48 V and E_p_ = −0.94 V (for L_2_). The oxidation potential of the metal center in [RuL_1_L_2_(NCS)_2_] is more negative, relative to those of the ligands, indicative of the electron-donating nature of the nitrogen to the ruthenium metal center. As expected, the irreversible oxidation peak at +0.63 V (Process **V**) was attributed to Ru(III)/Ru(II) [32]. The redox wave of the complex [RuL_1_L_2_(NCS)_2_] were observed at E_1/2_ = −0.86, −0.45 and −0.27 V for processes **I**, **II** and **III**, respectively.

In other to obtain a more accurate measurement of redox equivalency, the three reduction processes were studied by chronocoulometry using the equation:

$$Q=\frac{2{nFACD}^{\frac{1}{2}}t^{\frac{1}{2}}}{\pi^{\frac{1}{2}}}$$

**Figure 5:**
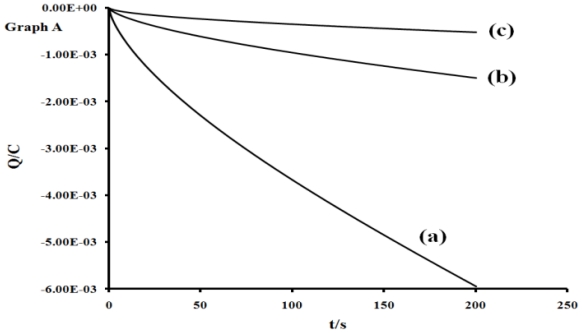

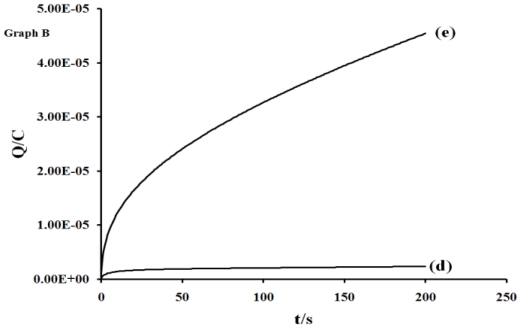
Plots of charge *vs.* time response for processes I, II and III; and IV and V (line a-e, respectively). Scan rate: 200 m Vs^−1^.

**Figure 6:**
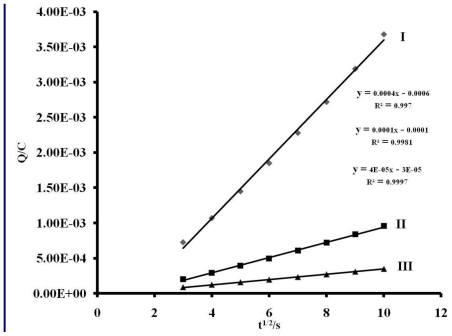

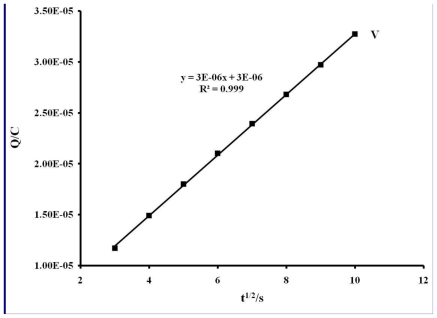
Anson plot of charge *vs*. square root of time (s) for processes I, II and III, and V. scan rate: 200 m Vs^−1^.

From the slope of the data when the quantity of electricity was plotted against square root of time (Figures 5 and 6), the number of electrons (n) for processes **I**, **II** and **III** were found to be in ratio (3:2:1), suggesting that these processes are multi-electronic in nature while process **IV** or **V** is a singleelectron process. This result indicates that different electron transfer processes are involved between the ruthenium ion, phenanthrolyl and the anthracenyl groups [55–57].

## 3. Experimental Section

### 3.1. Materials and General Physical Measurements

All chemical and reagents were analytically pure and used without further purification. 4,7-Dibromo-1,10-phenanthroline was synthesized as described in the literature [35]. 4,7-bis(2,3-dimethylacrylic acid)-1,10-phenanthroline and 4,7-bis(2,3-dimethylacrylic acidtrianthracenyl)- 1,10-phenanthroline were synthesized with slight modifications [33] (Scheme 1). All thin layer chromatography (TLC) analyses were done with aluminium sheet precoated with normal phase silica gel 60 F_254_ (Merck, 0.20 mm thickness) unless otherwise stated. The TLC plates were developed using any of the following solvent systems: Solvent system A: Dichloromethane-Methanol (9:1); Solvent system B: Dichloromethane-Methanol (7:3); Solvent system C: Dichloromethane- Benzene (3:7); Solvent system D: Chloroform-Methanol (1:1). Gel filtration was performed using Sephadex LH-20 previously swollen in specified solvent (s) prior to loading of extract onto the column (3.5 cm × 8.5 cm).

Melting points were determined using a Gallenkamp electrothermal melting point apparatus. Microanalyses (C, H, N, and S) were carried out with a Fisons elemental analyzer and Infrared spectra were obtained with KBr discs or nujol on a Perkin Elmer System 2000 FT-IR Spectrophotometer. UV-Vis and fluorescence spectra were recorded in a 1 cm path length quartz cell on a Perkin Elmer Lambda 35 spectrophotometer and Perkin Elmer Lambda 45 spectrofluorimeter, respectively. ^1^H and ^13^C Nuclear Magnetic Resonance (NMR) spectra were run on a Bruker EMX 400 MHz spectrometer for ^1^H and 100 MHz for ^13^C. The chemical shift values were reported in parts per million (ppm) relative to (TMS) as internal standard. Chemical shifts were also reported with respect to CDCl_3_ at δ_c_ 77.00 and δ_H_ CDCl_3_ at 7.25; and DMSO d_6_ at δ_c_ 40.98 and DMSO d_6_ at δ_H_ 2.50 for synthesized ligands and complexes. All electrochemical experiments were performed using Autolab potentiostat PGSTAT 302 (EcoChemie, Utrecht, The Netherlands) driven by the general purpose Electrochemical System data processing software (GPES, software version 4.9). Square wave voltammetric analysis was carried out at a frequency of 10 Hz, amplitude: 50 mV and step potential: 5 mV. A conventional three-electrode system was used. The working electrode was a bare glassy carbon electrode (GCE), Ag|AgCl wire and platinum wire were used as the pseudo reference and auxiliary electrodes, respectively. The potential response of the Ag|AgCl pseudo-reference electrode was less than the Ag|AgCl (3 M KCl) by 0.015 ± 0.003 V. Prior to use, the electrode surface was polished with alumina on a Buehler felt pad and rinsed with excess millipore water. All electrochemical experiments were performed in freshly distilled dry DMF containing TBABF_4_ as supporting electrolyte.

### 3.2. Synthesis of Ligand *1*

One hundred and fifty milligrams (4.84 mmol) of 4,7-dibromo-1,10-phenanthroline and *trans*-2,3-dimethylacrylic acid (0.97 g, 9.68 mmol) were dissolved in methanol (50 mL) and triethylamine (1.0 mL) and 20 mg palladium carbide were added. After 24 h reflux, the mixture gave a red solution, which was allowed to cool to room temperature and later concentrated under reduced pressure to afford red-brownish liquid product. To the crude product, 50 mL degassed water was added to form a homogenous phase solution, which was then extracted exhaustively with chloroform. The chloroform extract was concentrated *in vacuo* and then recrystallized in diethyl ether to afford L_1_. (White creamy solid, Yield, 1.98 g, 80.16 %; Mp: 85–86 °C). Selected data of ligand L_1_: ^1^H NMR (400 MHz, DMSO): δ 9.09 (d, *J* = 3.6 Hz, H-9), 8.55 (br, H-2), 8.43 (d, *J* = 8.0 Hz, H-8), 7.91 (s, H-5, H-6), 7.72 (d, J = 4.4 Hz, H-3), 2.54 (s, CH_3_), 1.70 (t, CH_3_). ^13^C NMR (400 MHz, DMSO): δ 169.67, 150.74, 146.40, 137.01, 129.30, 127.47, 124.10, 14.90, 12.75. Elemental analysis: Found: C 70.56, H 4.99, N 7.23; Calcd for C_24_H_20_N_2_O_4_: C 70.20, H 5.36, N, 7.44.

### 3.3. Synthesis of Ligand *2*

Fifty-eight milligrams (1.10 mmol) of 9-bromo-10-(2,3-dimethylacrylic acid)-dianthracene and 4,7-bis(9-bromoanthracenyl)-1,10-phenanthroline (0.37 g, 0.50 mmol) were dissolved in ethanol (40 mL) and stirred mechanically for about 25 min. To this solution, triethylamine (1 mL) and 0.05 g of palladium/carbide were added. The mixture was refluxed at 120 °C for 8 h. The crude product was filtered hot, allowed to cool to room temperature and the solvent removed by evaporation under reduced pressure. The pure product L_2_ was obtained by recrystallization in 50 % ethanol-ether mixture to afford reddish-brown solid (Yield 0.51 g, 54.2 %, Mp: 188–190 °C). Selected data of ligand L_2_: ^1^H NMR (400 MHz, CDCl_3_): δ 9.26 (d, *J* = 4.4 Hz, 2H), 8.40 (s, 2H), 7.67 (d, *J* = 4.4 Hz, 2H), 8.29 (dd, *J* = 3.4, 5.8 Hz, 4H), 7.98 (dd, *J* = 3.2, 6.4 Hz, 4H), 7.77 (dd, *J* = 3.8, 7.0 Hz, 4H), 7.45 (dd, *J* = 3.1, 6.6, 4H), 1.82 (s, CH_3_), 1.79 (s, CH_3_). ^13^C NMR (100 MHz, CDCl_3_): δ; 183.1, 149.9, 144.9, 139.5, 136.7, 134.1, 133.5, 131.6, 128.7, 128.1, 127.2, 126.6, 126.2, 125.3, 123.4, 14.6. IR data (KBr, cm^−1^): 3381, 3027, 2925, 1817, 1621, 1587, 1522, 1502, 1436, 1422, 1346, 1304, 1255, 1172, 1161, 1137, 1091, 1026, 960, 883, 778, 746, 623, 578. λ_max_/nm (ɛ/M^−1^cm^−1^) (Chloroform) 379 (41 000), 359 (42 900), 341 (38 260), 325 (14 750). Elemental analysis: Found: C 84.80, H 5.01, N 1.85; Calcd for C_106_H_68_N_2_O_4_.4H_2_O: C 84.55, H 5.09, N 1.86.

### 3.4. Synthesis of [Ru(II)-L_1_L_2_(NCS)_2_]

[RuCl_2_(dmso)_4_], used as metal precursor, was synthesized as reported [34]. The [RuL_1_L_2_(NCS)_2_] complex was synthesized following literature procedure [36] with slight modifications as follows: In a 250 mL flask, [RuCl_2_(dmso)_4_](0.05 g, 10.46 mmol) was dissolved in *N,N*-dimethylformamide followed by the successive addition of L_1_ (0.04 g, 10.46 mmol) and L_2_ (0.15 g, 10.46 mmol) and excess of NH_4_NCS (0.07 g, 10.46 mol). The mixture was heated at 120 °C for 5 h in the dark. The solution was allowed to cool to room temperature and filtered to remove unreacted starting material. The filtrate was concentrated to dryness and 40 mL of 0.05 M NaOH solution was added to give dirty brown precipitate which was filtered off. The pH of the resulting solution was adjusted to 3 with 0.5 M HNO_3._ The solution was left to stand in the fridge (−2 °C) for 12 h before being filtered and concentrated *in vacuo*. Water was added to the resulting semisolid to remove excess NH_4_NCS. The water insoluble product was collected on sintered glass crucible by suction filtration and washed with distilled water, followed by diethyl ether and dried. The resulting crude complex was purified twice by column chromatography on Sephadex LH 20 using chloroform-methanol (1:1) as eluting solvent (R_f_ = 0.53). The pure complex was isolated upon addition of 0.05 M HNO_3_ as a dark-brown solid (Yield 0.133 g, 42.4 %; Mp. 240–243 °C). Selected data for complex Ru-L_1_L_2_(NCS)_2_: ^1^H NMR (400 MHz, DMSO-d_6_): δ 9.38 (d, *J =* 14.80 Hz, 1H), 8.99 (d, s, 2H), 8.81 (d, *J =* 2.00 Hz, 2H), 8.70 (dd, *J =* 8.41, 1.87 Hz, 2H), 8.66 (d, *J =* 7.98 Hz, 3H), 8.46 (d, *J =* 8.49 Hz, 3H), 8.30 (d, *J =* 3.61 Hz, 3H), 8.22 (dd, *J =* 5.53, 3.36 Hz, 9H), 7.94 (dd, *J =* 5.47, 3.30 Hz, 14H), 7.88 (d, *J =* 8.66 Hz, 4H), 1.23 (s, CH_3_); ^13^C NMR: δ 183.2, 134.9, 134.1, 133.5, 130.8, 130.4, 129.7, 129.6, 128.9, 128.7, 128.4, 127.3, 126.3, 123.0, 122.6, 122.1, 121.7, 121.4. Elemental analysis: Found: C 77.23, H 4.73, N 4.34; Calcd. for: C_130_H_88_N_6_S_2_O_8_Ru. C 77.02, H 4.38, N 4.15.

## 4. Conclusions

A heteroleptic Ru(II) complex of phenanthroline containing oligo-anthracenyl carboxylic acid moieties have been synthesized and characterized by elemental analyses and spectroscopic techniques. The complex showed good visible absorption and high molar extinction coefficient. The intense emission is a significant contribution to the excited state from an interaction between the metal d-orbital and the ligand π-systems [58]. The electrochemical properties of the complex show significantly a single- to multi-electronic redox activity. The redox potential is far less positive than that reported for [Ru(bpy)_3_]^3+/2+^ or [Ru(tpy)_2_]^3+/2+^, which gave indication that it can undergo stable redox reaction. In the future, we focus on establishing the potentiality of the complex as a sensitizer for dye-sensitized solar cells (DSSCs)[59–61].

## Supporting Information

FT-IR, UV-Vis, ^1^H, ^13^C NMR spectra of L_1_ and L_2_ and the [RuL_1_L_2_(NCS)_2_] complex are included.

## Figures and Tables

**Table 1:** Cyclic voltammetric data for L_1_, L_2_ and [RuL_1_L_2_(NCS)_2_].

Compound	E_pa_/V	E_1/2_/V
L_1_	0.45	−0.23, −0.59
L_2_	0.48	−0.38, −0.57, 0.94
[RuL_1_L_2_(NCS)_2_]	0.17, 0.63	−0.27, −0.45, −0.86
